# Characterization of Diabetic Striatopathy With Repeated Follow-Up Using Multiple Imaging Studies

**DOI:** 10.7759/cureus.52223

**Published:** 2024-01-13

**Authors:** Ayumi Nishimura, Tomonobu Kado, Kazuyuki Tobe

**Affiliations:** 1 First Department of Internal Medicine, Graduate School of Medicine and Pharmaceutical Science, University of Toyama, Toyama, JPN

**Keywords:** neuroimaging, diabetes mellitus, hyperglycemic non-ketotic hemiballism, hyperglycemic non-ketotic hemichorea, diabetic chorea, diabetic striatopathy

## Abstract

Diabetic striatopathy is a rare condition with a prevalence of less than one in 100,000. Herein, we report a case of diabetic striatopathy exacerbated by hyperglycemia and hypoglycemia, with repeated follow-up with multiple imaging studies. This case suggested that putamen neuronal loss and dysfunction, gliosis, and ischemia are associated with diabetic striatopathy pathophysiology. In addition, striatal hyperintensity on T1-weighted MRI images was more pronounced after symptom remission when evaluated several times over a short period. Therefore, clinicians should be aware that even if MRI findings are normal in the very early stages of the onset of diabetic striatopathy, repeating MRIs at intervals may reveal typical findings.

## Introduction

Diabetic striatopathy is a rare disease that causes chorea or ballism, primarily in patients with type 2 diabetes mellitus and poor glycemic control [1-3]. Although diabetic hemiballism/hemichorea, hyperglycemic non-ketotic hemichorea/hemiballism, and other names have been used to describe this disease, diabetic striatopathy has only recently been used to describe the disease [1-5]. The term diabetic striatopathy is a nomenclature that focuses on imaging findings rather than symptoms [4]. Striatal hyperintensity on T1-weighted magnetic resonance imaging (MRI) is a characteristic of diabetic striatopathy [1-6]. However, the relationship between the imaging findings and the clinical course of the disease is not clearly understood. We report a case of first onset and subsequent recurrence of diabetic striatopathy in which follow-up of blood glucose changes, symptoms, and imaging findings using several modalities was traceable.

## Case presentation

**Figure 1:**
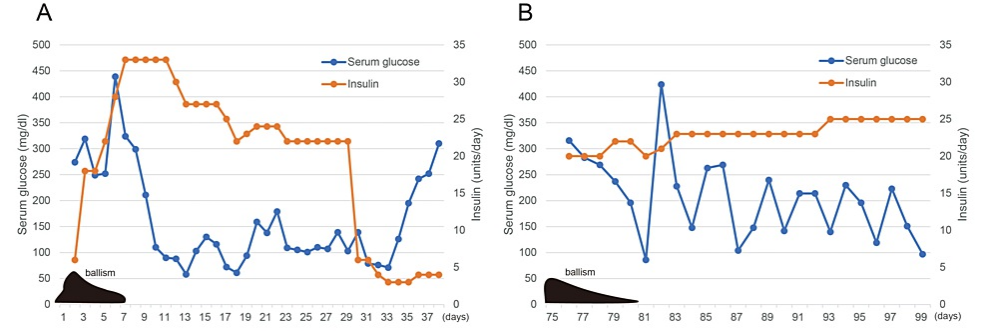
Clinical course Blood glucose levels, the amount of insulin administered, and the course of symptoms after admission are described. The number of days was counted from the date of first admission.

A 72-year-old man presented to our emergency department with thirst, polydipsia, polyuria, and difficulty in walking. He was diagnosed with type 2 diabetes mellitus at 53 years of age and started on insulin therapy at 63 years of age. Insulin therapy was discontinued at the age of 70 owing to frequent hypoglycemia, and alogliptin (50 mg/day) was started. Although he was diagnosed with hypertension, he did not take any antihypertensive medications. He interrupted his diabetes treatment at 71 years of age. At his visit to our hospital, his serum glucose level was 841 mg/dL, and glycosylated hemoglobin (HbA1c) was 16.3%. He presented with a temperature of 37.6° C, blood pressure of 173/53 mmHg, and oxygen saturation of 98% on room air. Consciousness was clear. Dyskinesia of the mouth and slight ballism of both lower limbs were observed. The cranial nerve examination was unremarkable. Resting tremors, muscle fiber spasms, and meningeal irritation were not observed. A Mini-Mental State Examination score of 27/30 indicated mild cognitive impairment. The patient tested negative for anti-glutamic acid decarboxylase antibodies. Serum C-peptide level had decreased to 0.7 ng/mL. The arterial blood pH was 7.412; no acidosis was observed; serum ketones were slightly elevated (total ketone bodies of 0.416 mmol/L, acetoacetic acid of 0.138 mmol/L, and 3-hydroxyacetic acid of 0.278 mmol/l), and urine ketones were negative. Mild renal impairment with a creatinine of 1.0 mg/dL and an estimated glomerular filtration rate of 59.6 mL/min/1.73 m^2^ were observed. The patient's clinical course is shown in Figure 1.

A summary of the imaging findings is presented in Table 1.

**Table 1:** A summary of imaging findings FLAIR - fluid-attenuated inversion recovery; DWI - diffusion-weighted imaging; ADC - apparent diffusion coefficient; SWI - susceptibility-weighted imaging; MRA - magnetic resonance angiography; MRS - magnetic resonance spectroscopy; SPECT - single-photon emission computed tomography; NAA/Cr - N-acetylaspartate/creatine; Cho/Cr - choline/creatine ratio

Modality	Typical case	①	②	③	④	⑤	⑥	⑦
CT	Striatal hyperdensity	Bilateral putamen hyperdensity	Bilateral putamen hyperdensity	Bilateral putamen hyperdensity	-	-	-	-
MRI								
T1-weighted	Striatal hyperintensity	Left putamen hyperintensity	Bilateral putamen hyperintensity	Bilateral putamen hyperintensity	Bilateral putamen hyperintensity	Left putamen hyperintensity	Left putamen hyperintensity	No signal abnormalities
T2-weighted	Variable	No signal abnormalities	Bilateral putamen hyperintensity	Bilateral putamen hyperintensity	Bilateral putamen hyperintensity	Bilateral putamen hyperintensity	Left putamen hyperintensity	-
FLAIR (striatum)	Variable	No signal abnormalities	Left putamen hyperintensity	Left putamen hyperintensity	Left putamen hyperintensity	No signal abnormalities	No signal abnormalities	No signal abnormalities
FLAIR (white matter)	No report	White matter hyperintensity	White matter hyperintensity	White matter hyperintensity	White matter hyperintensity	White matter hyperintensity	White matter hyperintensity	White matter hyperintensity
DWI	Variable	Bilateral putamen hypointensity	Bilateral putamen hypointensity	Bilateral putamen hypointensity	Bilateral putamen hypointensity	Bilateral putamen hypointensity	Bilateral putamen hypointensity	Bilateral putamen hypointensity
ADC	Variable	-	Left putamen hyperintensity	Left putamen hyperintensity	Left putamen hyperintensity	Left putamen hyperintensity	Left putamen hyperintensity	Left putamen hyperintensity
T2*	Variable	No signal abnormalities	No signal abnormalities	-	No signal abnormalities	No signal abnormalities	No signal abnormalities	-
SWI	Variable	-	-	-	Bilateral striatal hypointensity	Bilateral striatal hypointensity	Bilateral striatal hypointensity	-
MRA	Variable	No abnormality	-	-	-	-	-	No abnormality
MRS	Putamen and/or caudate nucleus NAA/Cr↓, Cho/Cr↑	Left putamen NAA/Cr↓	Left putamen NAA/Cr↓	Left putamen NAA/Cr↓, Cho/Cr↑	-	-	-	-
SPECT	Basal ganglia hypoperfusion	Left putamen hypoperfusion	-	-	-	-	-	.

**Figure 2:**
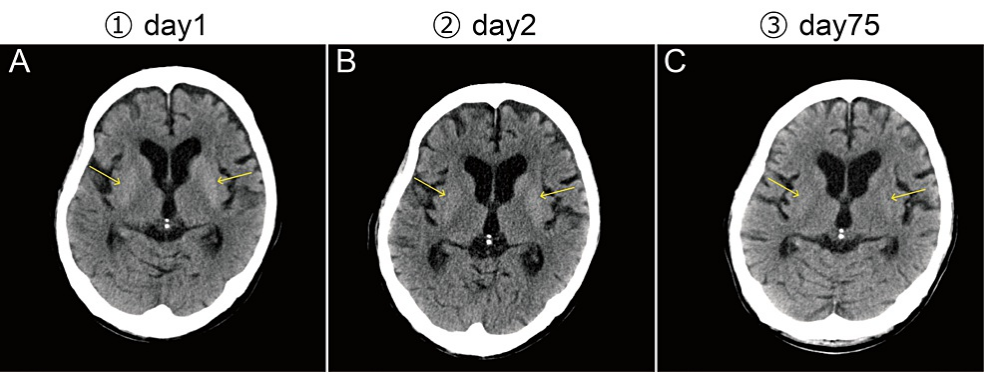
CT images CT showed hyperdensity in the bilateral putamen (arrow). On day 75, hyperdensity in the bilateral putamen was attenuated compared with that on days 1 and 2. The number of days was counted from the date of first admission.

CT on the day of admission showed hyperdensity in the bilateral putamen; however, no cerebral hemorrhage or brain tumor was observed (Figure 2A).

**Figure 3:**
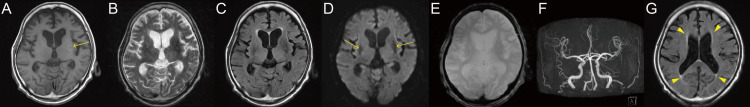
MRI images on the fourth day A: T1-weighted; B: T2-weighted; C: FLAIR (striatum); D: DWI; E: T2*; F: magnetic resonance angiography; G: FLAIR (white matter) Arrows indicate the area in which the signal changed in the putamen. Arrowheads indicate the area in which the signal changed in the white matter. The number of days was counted from the date of first admission. Table 1 summarizes these findings. FLAIR - fluid-attenuated inversion recovery; DWI - diffusion-weighted imaging

Insulin therapy was initiated on admission. Insulin sensitivity was better than expected, and the serum glucose level decreased to 56 mg/dL on the day of admission, resulting in hypoglycemia. On the second day, severe ballism appeared in both upper and lower limbs, predominantly in the right lower limb. CT showed hyperdensity in the bilateral putamen and no evidence of cerebral edema (Figure 2B). Diabetic striatopathy was suspected because of the sudden onset of bilateral ballism and hyperglycemia; the symptoms became more severe after a rapid change in serum glucose. We suspected cerebrovascular disease (e.g., subthalamic nucleus, striatum, globus pallidus, thalamus, and deep parietal lobe), aceruloplasminemia, Wilson's disease, Wernicke's encephalopathy, systemic lupus erythematosus (central nervous system lupus), and viral encephalopathy as differential diseases that might cause chorea and ballism. On the third day, risperidone was administered to treat involuntary movements and restlessness. On the fourth day, MRI showed hyperintensity in the left putamen on T1-weighted images (Figure 3A).

**Figure 4:**
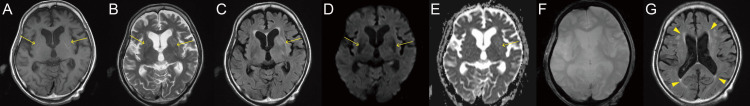
MRI images on day 16 A: T1-weighted; B: T2-weighted; C: FLAIR (striatum); D: DWI; E: ADC; F: T2*; G: FLAIR (white matter) Arrows indicate the area in which the signal changed in the putamen. Arrowheads indicate the area in which the signal changed in the white matter. The number of days was counted from the date of first admission. Table 1 summarizes these findings. FLAIR - fluid-attenuated inversion recovery; DWI - diffusion-weighted imaging; ADC - apparent diffusion coefficient

Dyskinesia of the mouth and ballism in both the upper and lower limbs resolved on the seventh day with risperidone and glycemic control. The patient started rehabilitation on the 10th day and could walk independently on the 15th day. MRI on the 16th day showed bilateral putamen hyperintensity on T1-weighted images (Figure 4A). 

**Figure 5:**
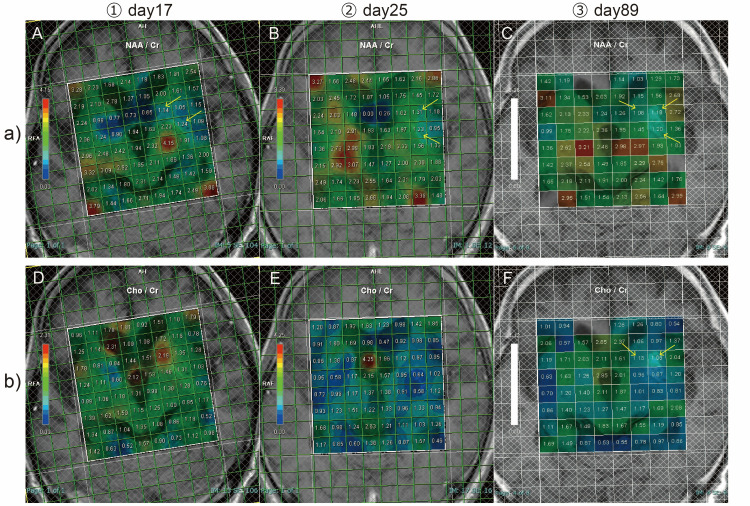
Magnetic resonance spectroscopy (MRS) images a: N-acetylaspartate/creatine ratio (NAA/Cr ratio) evaluated using MRS; b: choline/creatine ratio (Cho/Cr ratio) evaluated using MRS Arrows indicate the area where the signal changed in the putamen. The number of days was counted from the date of first admission.

Magnetic resonance spectroscopy (MRS) on the 17th day showed decreased N-acetylaspartate/creatine ratio (NAA/Cr ratio) in the left putamen (Figure 5A).

**Figure 6:**
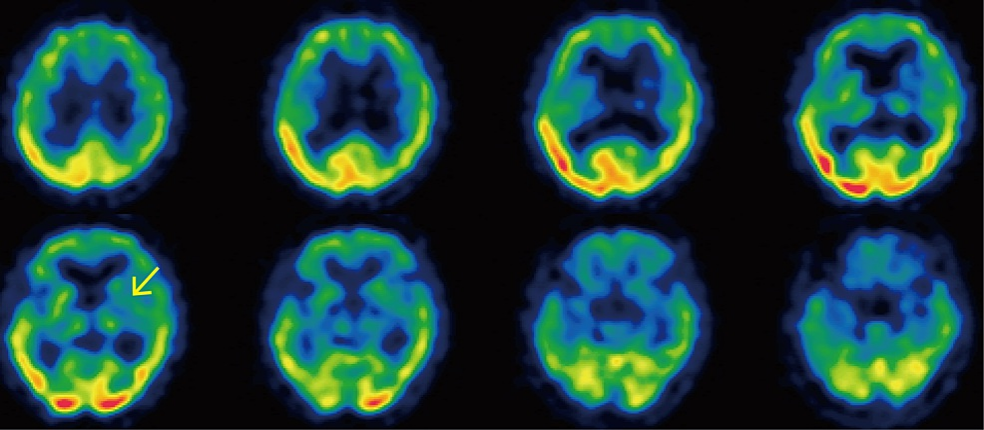
SPECT images SPECT showed hypoperfusion in the left putamen (arrow). The number of days was counted from the date of first admission. SPECT - single-photon emission computed tomography

Single-photon emission computed tomography (SPECT) on the 18th day showed hypoperfusion in the left putamen (Figure 6).

**Figure 7:**
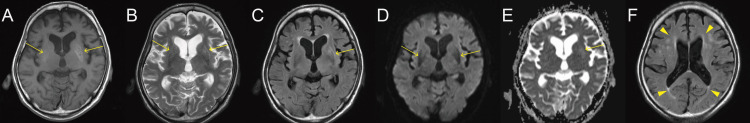
MRI images on day 25 A: T1-weighted; B: T2-weighted; C: FLAIR (striatum); D: DWI; E: ADC; F: FLAIR (white matter) Arrows indicate the area in which the signal changed in the putamen. Arrowheads indicate the area in which the signal changed in the white matter. The number of days was counted from the date of first admission. Table 1 summarizes these findings. FLAIR - fluid-attenuated inversion recovery; DWI - diffusion-weighted imaging; ADC - apparent diffusion coefficient

MRI on the 25th day showed bilateral putamen hyperintensity on T1-weighted images more clearly than on the fourth day and the 16th day (Figure 7A), and MRS showed a decreased NAA/Cr ratio in the left putamen (Figure 5B) on the 25th day. By this time, he could walk long enough to go shopping alone at the hospital's convenience store. Continuing frequent insulin injections after discharge was considered impossible because of the patient's lack of awareness of his illness. As insulin injections require family supervision, vildagliptin was administered on the 23rd day, and repaglinide was administered on the 30th day, switching from multiple daily insulin injection therapy to basal-supported oral therapy.

**Figure 8:**
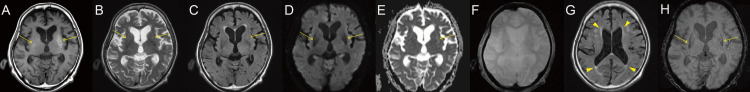
MRI images on day 37 A: T1-weighted; B; T2-weighted; C: FLAIR (striatum); D: DWI; E: ADC; F: T2*; G: FLAIR (white matter); H: SWI Arrows indicate the area in which the signal changed in the putamen. Arrowheads indicate the area in which the signal changed in the white matter. The number of days was counted from the date of first admission. Table 1 summarizes these findings. FLAIR - fluid-attenuated inversion recovery; DWI - diffusion-weighted imaging; ADC - apparent diffusion coefficient; SWI - susceptibility-weighted imaging

MRI on the 37th day showed bilateral putamen hyperintensity on T1-weighted images that were more pronounced than those on the 25th day (Figure 8A).

**Figure 9:**
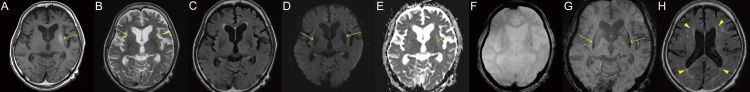
MRI images on day 87 A: T1-weighted; B: T2-weighted; C: FLAIR (striatum); D: DWI; E: ADC; F: T2*; G: SWI; H: FLAIR (white matter) Arrows indicate the area in which the signal changed in the putamen. Arrowheads indicate the area in which the signal changed in white matter. The number of days was counted from the date of first admission. Table 1 summarizes these findings. FLAIR - fluid-attenuated inversion recovery; DWI - diffusion-weighted imaging; ADC - apparent diffusion coefficient; SWI - susceptibility-weighted imaging

The patient was discharged on the 38th day. At a follow-up visit one month after discharge, dyskinesia of the mouth and predominant right lower limb ballism were observed. At that time, serum glucose level was 687 mg/dL, and HbA1c was 13.7%, and he was admitted to the hospital for the second time on the 75th day. The CT showed hyperdensity in the bilateral putamen (Figure 2C), which was attenuated compared with that at the time of the first admission. MRI showed hyperintensity in the left putamen on T1-weighted images (Figure 9A).

**Figure 10:**
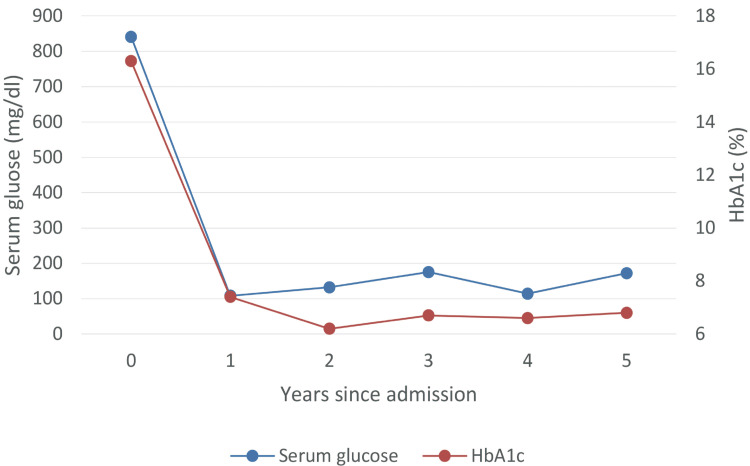
Course of blood glucose and HbA1c Glycemic control was good after the second admission, and no recurrence of diabetic striatopathy was observed. HbA1c - glycosylated hemoglobin

With infusion and frequent insulin therapies, the serum blood glucose level gradually improved, and involuntary movements disappeared on the 80th day. MRS performed on the 89th day showed a decrease in the NAA/Cr ratio and increased the Cho/Cr ratio in the left putamen, suggesting neuronal damage and gliosis (Figures 5C-F). As hyperglycemia was suspected of having caused recurrent diabetic striatopathy, we judged that treatment with frequent insulin injections was essential; with the cooperation of the family, the patient was discharged on the 99th day with multiple daily insulin injections. With continued frequent insulin injections, the patient maintained good glycemic control after discharge and showed no recurrence of diabetic striatopathy (Figure 10).

**Figure 11:**
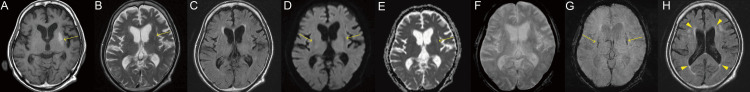
MRI images on day 125 A: T1-weighted; B: T2-weighted; C: FLAIR (striatum); D: DWI; E: ADC; F: T2*; G: SWI; H: FLAIR (white matter) Arrows indicate the area in which the signal changed in the putamen. Arrowheads indicate the area in which the signal changed in white matter. The number of days was counted from the date of first admission. Table 1 summarizes these findings. FLAIR - fluid-attenuated inversion recovery; DWI - diffusion-weighted imaging; ADC - apparent diffusion coefficient; SWI - susceptibility-weighted imaging

MRI on the 125th day showed hyperintensity in the left putamen on T1-weighted images (Figure 11A).

**Figure 12:**
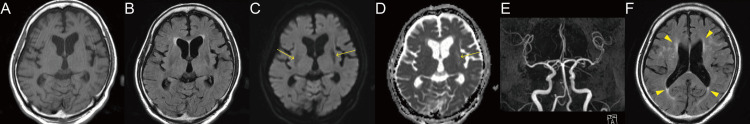
MRI images taken five years after the onset of diabetic striatopathy A: T1-weighted; B; FLAIR (striatum); C: DWI; D: ADC; E: MRA; F: FLAIR (white matter) Arrows indicate the area in which the signal changed in the putamen. Arrowheads indicate the area in which the signal changed in white matter. The number of days was counted from the date of first admission. Table 1 summarizes these findings. FLAIR - fluid-attenuated inversion recovery; DWI - diffusion-weighted imaging; ADC - apparent diffusion coefficient; MRA - magnetic resonance angiography

Five years after the first onset of diabetic striatopathy, the high-intensity signal in the left putamen on the T1-weighted MRI disappeared (Figure 12A).

## Discussion

Diabetic striatopathy has a high incidence in older Asian women and patients with poorly controlled type 2 diabetes mellitus. At first onset, ketones are negative, and involuntary movements are often unilateral [1-3]. Our patient was an older Asian male with poorly controlled type 2 diabetes mellitus. At the first onset, our patient had negative urinary ketones but mildly elevated blood ketones and bilateral involuntary movements. Impaired renal function is a risk factor for the development of diabetic striatopathy [7], and mild renal impairment has been reported in this case. In addition, an exacerbation of involuntary movements with a sudden drop in blood glucose levels after hospitalization was observed. Once the symptoms disappeared, the patient developed diabetic striatopathy again due to hyperglycemia. The symptoms at the recurrence time were unilateral, unlike those at the initial onset.

CT typically shows striatal hyperdensity [1,8], especially in the putamen and caudate nucleus contralateral to the involuntary movements. Most reports indicate that striatal hyperdensity on CT fades within a month and disappears within two months [3]. In this case, CT at the time of the second admission showed that the hyperdensity in the bilateral putamen had diminished compared with that at the time of the first admission; we speculate that the image change may have captured the process in which the hyperdensity that occurred at the time of the first admission was fading away. On MRI, T2-weighted, FLAIR, DWI, T2*, and SWI findings vary, although T1-weighted images typically show striatal hyperintensity [1,8-12]. As in previous reports, striatal hyperintensity on T1-weighted MRI was the clearest diagnostic indicator of diabetic striatopathy in this case. However, the findings were more apparent after a longer interval than at the initial onset. Striatal hyperdensity on CT and striatal hyperintensity on T1-weighted MRI have been reported to be typical of any episode of hyperglycemia or hypoglycemia [1,13]. There is no consensus on the characteristics of imaging findings in ketosis [14]. In this case, CT showed hyperdensity in the bilateral putamen during hyperglycemia, and the same findings were seen immediately after transient hypoglycemia.

In summary, the CT imaging findings did not change within a short period of time, even after a hypoglycemic episode following hyperglycemia. In addition, MRI after hyperglycemia and subsequent hypoglycemic episodes showed hyperintensity in the left putamen on T1-weighted images. Based on the findings of this case alone, it is difficult to distinguish the effects of hyperglycemia, hypoglycemia, and ketosis on imaging findings, and further studies are needed to differentiate them. MRS is a testing method that uses nuclear magnetic resonance to noninvasively measure trace amounts of substances in living organisms. MRS findings in diabetic striatopathy are a decrease in the NAA/Cr ratio, which reflects neuronal injury or dysfunction in the putamen or caudate nucleus, and an increase in the Cho/Cr ratio, which reflects gliosis [15,16]. In the present case, a decrease in the NAA/Cr ratio was observed at the time of initial admission, and an increase in the Cho/Cr ratio was observed at the time of recurrence. Hypoperfusion in the basal ganglia, contralateral to the involuntary movements, is typical on SPECT [17,18] and was also observed in our patient. Although decreased gamma-aminobutyric acid (GABA) levels and perfusion in the basal ganglia have been reported as factors in the pathogenesis of diabetic striatopathy [1], the MRS and SPECT findings of this case suggest that neuronal loss and dysfunction, gliosis, and ischemia in the putamen are associated with the pathophysiology of diabetic striatopathy.

Moreover, in our case, white matter hyperintensities were observed on MRI FLAIR, a characteristic finding of small vessel disease [19], which is particularly common in older people and has been reported to correlate with reduced perfusion in the middle cerebral artery [20]. Decreased perfusion of blood vessels innervating the basal ganglia (such as lenticulostriate arteries branching from the middle cerebral artery) may be a factor in the appearance of involuntary movements, suggesting that older patients with white matter hyperintensity on MRI FLAIR are more likely to develop diabetic striatopathy, although additional studies are needed.

To the best of our knowledge, no report has repeatedly evaluated cases of diabetic striatopathy using multiple imaging modalities over a short period. Diabetic striatopathy has a good prognosis, and invasive biopsy is rarely performed. Because of the difficulty in obtaining histological findings, repeated follow-ups with multiple imaging studies help reveal the pathophysiology.

## Conclusions

The course of this case suggests that hyperglycemia, antidiabetic-induced sudden hypoglycemia, and rapid changes in serum blood glucose levels exacerbate diabetic striatopathy. Avoiding rapid changes in serum blood glucose levels, particularly in poorly controlled and older patients with type 2 diabetes, may help prevent the onset or exacerbation of diabetic striatopathy. Striatal hyperintensity on T1-weighted MRI images, which is considered typical, showed a mild change immediately after the onset of diabetic striatopathy and became more pronounced even after symptom remission than immediately after onset. Clinicians should be aware that even if MRI findings are normal in the early stages of disease onset, repeating MRIs at intervals may reveal typical findings.
